# Retraction: Dual generative adversarial networks based on regression and neighbor characteristics

**DOI:** 10.1371/journal.pone.0324676

**Published:** 2025-05-15

**Authors:** The PLOS One Editors

The *PLOS One* Editors retract this article [1] due to concerns about:

- Compliance with PLOS policies on Submission and Publication of Related Studies.
- Similarities to a previous publication [2].
- The use of terminology that differs from standards in the field (e.g. “deep gaining knowledge” instead of “deep learning”).
- The reliability of the reported results and conclusions.

All authors agreed with retraction.

The retracted article [1] was removed from the *PLOS One* website at the time of retraction due to the similarities with [2]. The article’s Copyright and Data Availability statements were also updated at the time of retraction, and the removed contents are no longer offered under the Creative Commons Attribution License.
